# Genome Sequence of the Group III *Clostridium botulinum* Strain Eklund-C

**DOI:** 10.1128/genomeA.00044-13

**Published:** 2013-03-14

**Authors:** Karl A. Hassan, Sasha G. Tetu, Liam D. H. Elbourne, Eric A. Johnson, Ian T. Paulsen

**Affiliations:** a Department of Chemistry and Biomolecular Sciences, Macquarie University, NSW, Australia; b Department of Bacteriology, Botulinum Toxins Laboratory, University of Wisconsin, Madison, Wisconsin, USA; c J. Craig Venter Institute, Rockville, Maryland, USA

## Abstract

The neurotoxins produced by *Clostridium botulinum* strains are among the world’s most potent toxins and are the causative agents of paralytic botulism. Here, we present the draft genome sequence of the group III *C. botulinum* strain Eklund-C, including a pseudolysogen-like bacteriophage that harbors the type C neurotoxin operon.

## GENOME ANNOUNCEMENT

*Clostridium botulinum* strains are formidable human and animal pathogens that produce botulinum neurotoxin (BoNT), the most potent neurotoxin described to date and the causative agent of botulism. Animal botulism cases are commonly associated with group III *C. botulinum* strains, which harbor type C or D BoNT genes. Here, we report the draft genome sequence of the prototypical *C. botulinum* group III BoNT/C-producing Eklund strain (Eklund-C). Genomic DNA was sequenced using a Sanger shotgun method to 8-fold coverage and was assembled and annotated as described previously (1). The assembled genome sequence contains 2.96 Mb of sequence data containing 2,578 putative protein-coding genes, 251 putative pseudogenes, and 126 structural RNA genes.

Group III *C. botulinum* strains are phylogenetically related to strains *Clostridium novyi* and *Clostridium haemolyticum* (2, 3). Genome sequences for two strains within this phylogenetic group have been published, those of *C. botulinum* C/D 08-BKT015925 (4) and *C. novyi* NT (5, 6). Approximately 75 to 78% of the annotated protein-coding genes or pseudogenes within the Eklund-C strain were conserved in the 08-BKT015925 and NT strains, that is, 2,206 and 2,132 genes or pseudogenes, respectively, as determined using tBLASTx 2.2.25+ (7) searches (*E* value ≤1*e* - 30). Furthermore, the majority of the Eklund-C scaffold sequences could be aligned to the 08-BKT015925 and NT chromosomal sequences using Mauve 2.3.1 (8), highlighting the synteny among the three genomes.

A primary differentiating factor between the toxigenic group III *C. botulinum* strains and closely related species, such as *C. novyi*, is their carriage of BoNT/C or BoNT/D genes on an episomal pseudolysogen-like prophage (9, 10). Five scaffolds containing a total of 177 kb of sequence data are predicted to comprise the Eklund-C BoNT/C bacteriophage genome, which can be aligned to the BoNT/C-encoding phage isolated from strains *C. botulinum* 08-BKT015925 (4) and *C. botulinum* C-Stockholm (11). The sequenced Eklund-C BoNT/C phage genome displays features of a circular self-replicating episomal element: (i) the scaffolds bear no similarity to the *C. novyi* NT chromosomal sequence, (ii) it contains only one copy of a putative terminal direct repeat sequence that is spanned by numerous sequencing reads and is predicted to mediate circularization of the lysogen, as with the c-st phage (11), and (iii) the two halves of the Eklund-C phage sequence display distinctly different gene orientations and GC skews that diverge abruptly at the putative replication origin and terminus.

The putative Eklund-C phage contains 198 predicted protein-coding genes or pseudogenes. Of these sequences, 135 and 99 were identified within the 08-BKT015925 and C-Stockholm phage genomes, respectively. Most conserved genes are found in syntenous blocks of sequence that are interspersed by novel regions encoding primarily hypothetical proteins. The most highly conserved phage genes include those within the BoNT operon (CBC_A0165-A0170), as well as several phage structural genes (12). BoNT/C- or BoNT/D-encoding phages typically carry a large number of insertion sequence (IS) elements (6). At least 12 intact or degraded IS elements were identified in the Eklund-C prophage, all of which belong to the IS*200/605* family. Most of these are related to the elements observed in the 08-BKT015925 and C-Stockholm phages; however, the locations and copy numbers of many of these elements are varied.

### Nucleotide sequence accession numbers.

The draft genome sequence for *C. botulinum* Eklund-C has been included in the GenBank Whole-Genome Shotgun (WGS) database under the accession no. ABDQ00000000. The version described is accession no. ABDQ00000000.1.
